# The cAMP response element modulator (CREM) regulates T_H_2 mediated inflammation

**DOI:** 10.18632/oncotarget.6041

**Published:** 2015-10-08

**Authors:** Eva Verjans, Kim Ohl, Lucy K. Reiss, Femke van Wijk, Antonaneta A. Toncheva, Anastasia Wiener, Yin Yu, Annette D. Rieg, Vincent D. Gaertner, Johannes Roth, Edward Knol, Michael Kabesch, Norbert Wagner, Stefan Uhlig, Christian Martin, Klaus Tenbrock

**Affiliations:** ^1^ Department of Pediatrics, Medical Faculty, RWTH Aachen, Aachen, Germany; ^2^ Institute of Pharmacology and Toxicology, RWTH Aachen, Aachen, Germany; ^3^ Department of Pediatric Immunology, University Medical Center Utrecht, Utrecht, Netherlands; ^4^ Department of Pediatric Pneumology and Allergy, University Children`s Hospital Regensburg (KUNO), Regensburg, Germany; ^5^ Department of Anaesthesiology, Medical Faculty, RWTH Aachen, Aachen, Germany; ^6^ Institute of Immunology, University of Münster, Münster, Germany; ^7^ Member of the German Lung Research Center (DZL), GieΔen, Germany

**Keywords:** allergic disease, asthmatic response, T cell dysregulation, transcription factor, T_H_2, Immunology Section, Immunity, Immune response

## Abstract

A characteristic feature of allergic diseases is the appearance of a subset of CD4^+^ cells known as T_H_2 cells, which is controlled by transcriptional and epigenetic mechanisms. We aimed to analyze the role of CREM, a known transcriptional activator of T cells, with regard to T_H_2 responses and allergic diseases in men and mice. Here we demonstrate that T cells of asthmatic children and PBMCs of adults with atopy express lower mRNA levels of the transcription factor CREM compared to cells from healthy controls. CREM deficiency in murine T cells results in enhanced T_H_2 effector cytokines *in vitro* and *in vivo* and CREM^−/−^ mice demonstrate stronger airway hyperresponsiveness in an OVA-induced asthma model. Mechanistically, both direct CREM binding to the *IL-4* and *IL-13* promoter as well as a decreased IL-2 dependent STAT5 activation suppress the T_H_2 response. Accordingly, mice selectively overexpressing CREMα in T cells display decreased T_H_2 type cytokines *in vivo* and *in vitro,* and are protected in an asthma model. Thus, we provide evidence that CREM is a negative regulator of the T_H_2 response and determines the outcome of allergic asthma.

## INTRODUCTION

Allergic diseases represent significant global health problems with dramatically increasing morbidity during the last decades. Allergies can affect people of all ages, however the prevalence in childhood [1] is up to 39% [2]. Allergic asthma is characterized by airway inflammation, recurrent bronchospasms, airway hyperresponsiveness (AHR) and mucus hypersecretion [3]. The asthmatic response develops as a crosstalk between innate cells like dendritic cells, NK cells and type 2 lymphoid cells (NK cells [4], type 2 innate lymphoid cells [5] and the adaptive immune system with T_H_2 cells becoming important mediators of allergic inflammation [6–8]. T_H_2 cytokines promote T and B cell proliferation and activation, B cell isotype switching as well as eosinophilia and AHR [8–10]. Differentiation towards T_H_2 cells is actively induced by polarizing signals which up-regulate the master regulator GATA3 [11]. IL-4 is the most important cytokine for T_H_2 differentiation since it induces phosphorylation and activation of STAT6, which is essential for GATA3 expression [12]. Once upregulated, GATA3 drives T_H_2 differentiation by activating its own expression in a cell autonomous manner. Differentiated T_H_2 effector cells produce cytokines like IL-4, IL-13 and IL-5 upon TCR restimulation [13]. Beyond this mechanism, IL-2 and IL-2 mediated phosphorylation of STAT5 are critically involved in the T_H_2 differentiation [14, 15]. During the early phase of T_H_2 differentiation IL-2 induces IL-4Rα expression and this facilitates enhanced T cell responsiveness to the surrounding IL-4 [16]. Additionally, STAT5a augments IL-4 production by altering chromatin accessibility at the IL4 gene locus in differentiated T_H_2 cells [14, 15]. Thus, IL-2 is critically involved in the T_H_2 cell priming, as well as in the maintenance of the differentiated state.

Aside from STAT5 and GATA3 several other transcription factors contribute to the regulation of T_H_2 differentiation and maintenance of T_H_2 cells, among others c-MAF, NOTCH, IRF4 and Dec2 [17]. It is therefore widely accepted that a complex regulatory network of transcription factors, as well as chromatin remodeling and epigenetic modifications ensure T_H_2 commitment and maintenance. Uncovering these mechanism becomes more important as recent studies suggest that T cell subsets are not all terminally differentiated cells and plasticity of human T cell subsets opens new perspectives for immune-modulatory therapies [8]. Thus, the identification of additional factors may help to further elucidate the mechanisms through which specific T cell lineage decisions are facilitated.

Genome-wide association studies and transcriptome analyses have been performed aiming to identify factors influencing different asthmatic phenotypes. Recently, the cAMP responsive element modulator (CREM) was published in a list of genes downregulated in CD4^+^ T cells of children with recurrent and persistent wheeze [18] without further investigation and discussion of the significance. So far CREM has only been discussed in the context of autoimmunity being an important molecule in the T cell pathogenesis of systemic lupus erythematosus [19]. CREMα, a CREM isoform generated by alternative splicing, has key functions as an epigenetic and transcriptional regulator of cytokine expression in T cells from SLE patients [19]. T cells from patients with systemic lupus erythematosus (SLE) exhibit CREMα overexpression [20]. CREMα contributes to silencing of *IL2* in these cells through *trans*-repression and tissue- and region-specific recruitment of specific DNA and histone methyltransferases or HDACs. [21]. CREM is a member of the ATF/CREB type bZip transcription factors family. Both CREB and CREM are activated by the second messenger cAMP, which is directly involved in the regulation of T_H_2 type cytokines such as IL-5, as well as in regulation of the IFNγ promoter activity by promoter methylation [22–24]. cAMP activates proteinkinase A that phosphorylates, and thus activates, CREB and CREM. Members of the ATF/CREB family bind to the cAMP-response element (CRE) in the promoter regions of target genes. This binding results in either suppression or activation of promoter activity, and respectively of gene expression [25, 26]. ATF3 has been shown to directly bind to IL-4, IL-5 and IL-13 promoters and it was suggested that this is one mechanism of ATF3 mediated T_H_2 regulation [27]. In addition, ATF3 binding sites were enriched in a transcriptomic and epigenomic analysis of primary human T cells in patients with asthma [28].

We aimed to analyze if the downregulation of CREM in CD4^+^ T cells of wheezing children has functional relevance for asthmatic diseases and wondered if CREM influences the differentiation of T_H_2 cells and consecutive asthmatic inflammation. To address this question we analyzed CREM expression in humans with atopy and CREM function in murine experimental asthma models and furthermore analyzed transcriptions mechanism of CREM in T_H_2 cell differentiation.

## RESULTS

### CREM expression is downregulated in asthma and allergy

**Figure 1:**
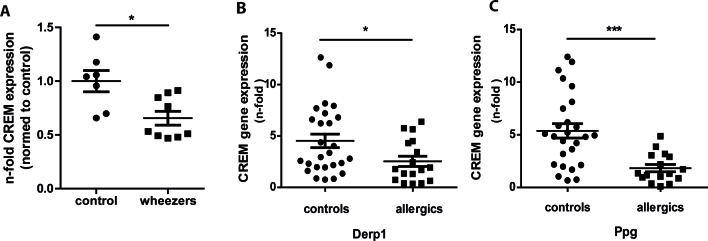
Expression of CREM is decreased in asthmatic children and allergic adults **a.** CD4^+^ T cells of children with persistent and intermittent wheeze (*n* = 10) and of healthy control children (*n* = 7) were isolated, RNA was extracted and levels of CREM were determined by qRT-PCR. Figure shows fold change of asthmatic children compared to controls. **b.**-**c.** PBMCS from adults with history of allergy (*n* = 17) and healthy controls (*n* = 26) were stimulated for 48h with house dust mite allergen Derp1 or peptidoglycane (Ppg), RNA was extracted and CREM levels were analyzed by qRT-PCR. Results show n-fold CREM expression normalized to unstimulated PBMC. All results represent means ± SEM; *p < 0.05, ***p < 0.001. See also supplementary Figures S1 and S2.

In a transcriptome analysis using unstimulated CD4^+^ T cells of children with recurrent and persistent wheeze *CREM* was found downregulated compared to healthy controls and published on a list of genes without further validation and discussion of the significance [18]. To further explore the relevance and to confirm the data, we evaluated *CREM* levels using cDNA from the same samples that were subjected to affymetrix analysis and measured *CREM* levels. As expected, *CREM* levels were significantly lower in CD4^+^ T cells from wheezing children compared to healthy controls (Fig. 1a). Next, we sought to confirm this in an independent cohort using PBMCs of 17 adults with a medical history of atopy and 26 controls. While overall *CREM* expression did not differ significantly between the two groups (supplementary Fig. S1a), stimulation with the house dust mite allergen Derp1 or with peptidoglycan (Ppg) for 48 hours resulted in a significantly decreased expression of *CREM* in the allergic cohort (Fig. 1b-1c), while unspecific stimulation with PHA only showed an insignificant tendency towards lower expression of CREM in the allergic cohort (supplementary Fig. 1b). In addition, *IL-4* and *CREM* mRNA expression showed a tendency towards negative correlation in the allergic cohort after stimulation with Derp1, while this was not the case in controls (supplementary Fig. S1c and S1d). In addition, to analyze whether CREM is also regulated during allergic sensitization in mice *in vivo*, we injected mice with ovalbumin and aluminum hydroxide (alu) followed by inhalation with ovalbumin. Our data indicated significantly lower mRNA and protein CREM levels in lung tissue after OVA-sensitization (supplementary Fig. S2a-S2c).

### Genetic deletion of CREM enhances the expression of T_H_2 cytokines and of IL-2

**Figure 2:**
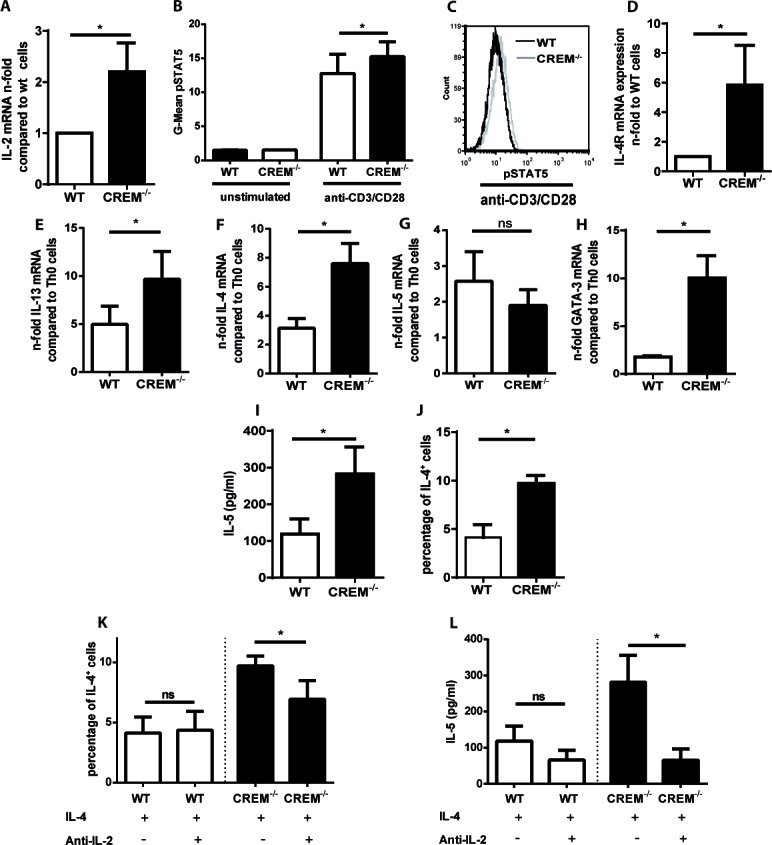
Genetic deletion of CREM enhances T_H_2 production in an IL-2-dependent manner **a.** CD4^+^ T cells from CREM^−/−^ and wt mice were stimulated with plate-bound anti-CD3 and anti-CD28 for 48 hours. RNA was isolated and qRT-PCR was performed with primers specific for IL-2. Results represent means of 5 experiments ± SEM. **b.** CD4^+^ T cells were simulated as in **a.** and pSTAT5-levels were measured by flow cytometry. Mean fluorescence intensity (MFI) values for pSTAT5 were determined. Bars show means of 3 experiments ± SEM. **c.** Representative histograms of pSTAT5. **d.** Unstimulated CD4^+^ T cells from CREM^−/−^ and wt mice were harvested, RNA was isolated and qRT-PCR was performed with primers specific for *Il4R*. Bars show n-fold levels of *Il4R* expression. **e.**-**h.** CD4^+^ T cells from CREM^−/−^ and wt mice were stimulated with plate-bound anti-CD3 and anti-CD28 in the presence of IL-4. qRT-PCR was performed with primers specific for *Il13* **e.**, *Il4* **f.**, *Il5* **g.** and *GATA3* **h.** Graphs show fold-change compared to T_H_0 cells, which were stimulated with plate-bound anti-CD3 and anti-CD28 in the absence of IL-4. Results represent means ± SEM of 3-5 experiments. **i.**-**l.** CD4^+^ T cells from CREM^−/−^ and wt mice were stimulated as in **e**. for 3-5 days. **i.** Cell supernatants were analyzed by IL-5-ELISA. Bars show concentrations of IL-5 ± SEM of 6 experiments. **j.** Intracellular IL-4 was measured by flow cytometry. Bars show percentages of IL-4^+^ cells ± SEM of 3 experiments. **k.** CD4^+^ T cells from CREM^−/−^ and wt mice were stimulated as in **e.** in the presence or absence of anti-IL-2. IL-4^+^ cells were determined as described in **j.**, *n* = 3 experiments. **l.** Cell supernatants from **k.** were analyzed by IL-5-ELISA, *n* = 6 experiments, results represent means ± SEM. *p < 0.05. See also supplementary Figure S3.

Having shown that CREM expression is reduced during allergic sensitization in different cohorts, we then explored the potential pathophysiological significance during T_H_2 differentiation. We previously demonstrated that CREMα overexpression in T cells obtained from SLE patients results in reduction of *IL-2* transcription, which can be restored by genetic silencing of CREMα [29]. Additionally, we generated a mouse overexpressing CREMα specifically in T cells (CD2CREMαtg mice) characterized by decreased expression of IL-2 and enhanced expression of IL-17 [30]. We therefore evaluated whether on the other hand the genetic deletion of CREM would result in elevated IL-2 expression and phosphorylation of STAT5. MACS isolated CREM^−/−^ CD4^+^ T cells showed enhanced *Il2* transcription 24 hours after stimulation with anti-CD3 and anti-CD28 (Fig. 2a). Accordingly, CREM^−/−^ T cells consistently displayed increased pSTAT5 levels (Fig. 2b-2c). It has been previously shown that T cell receptor stimulation induces IL-4Rα expression by an IL-2 and STAT5 dependent mechanism [16]. Enhanced expression of IL-4Rα promotes cellular responsiveness to IL-4 [16] and therefore is critically involved in the T_H_2 priming and differentiation. Furthermore, IL-2 signaling intensifies chromatin accessibility at the *Il-4* locus [14]. Thus, we then assessed transcription of *Il4r, Il4, IL5* and *Il13* in these T cells. A clear upregulation of *Il4r* was present already in unstimulated CREM^−/−^ T cells (Fig. 2d). When T cells were stimulated with anti-CD3 and anti-CD28, followed by addition of IL-4 to induce their polarization towards T_H_2 direction, we observed a striking, significantly higher upregulation of *Il4, Il13* and *Gata3* mRNA-levels in CREM^−/−^ T cells compared to wildytpe T cells, while *Il5* was not regulated on the mRNA level at that timepoint suggesting a different kinetic of transcription (Fig. 2e-2h). However, IL-5 protein levels were enhanced in CREM^−/−^ CD4^+^ T cells (Fig. 2i). Moreover, we counted significantly higher percentages of IL-4^+^ cells in the CREM^−/−^ T cells compared to wt T cells (Fig. 2j). Finally, increased levels of phosphorylated STAT6 in CREM^−/−^ T cells in T_H_2 polarizing conditions were detected (Supplementary Fig. S3), directly related to the enhanced expression of *Il4r* and dependent on IL-4 signaling [31]. The pSTAT6 differences seem modest but are consistently upregulated in CREM^−/−^ T cells.

Since CREM downregulates IL-2 expression, we blocked the enhanced IL-2 protein levels in CREM^−/−^ T cells by addition of anti-IL-2 in order to demonstrate that our previous results depend on the regulation by IL-2.

Indeed, after T_H_2 differentiation, the increased percentage (already shown in Fig. 2j) of IL-4^+^ CD4^+^ CREM^−/−^ T cells could be reduced by additional incubation with anti-IL-2 (Fig. 2k), while the percentage of these cells was stable in wt cultures. The same was observed for the IL-5 protein levels in the supernatant measured by ELISA (Fig. 2l). Thus, the effect of CREM on T_H_2 type cytokines secretion could be at least partially explained by CREM-mediated IL-2 regulation.

### Genetic deletion of CREM aggravates allergic airway hyperresponsiveness and inflammation

To address the pathophysiological significance of these findings CREM^−/−^ mice and appropriate wt animals were sensitized with OVA and subsequently lung function measurements were performed on day 35 after OVA challenges.

Sensitization itself slightly worsened the lung mechanics compared to Alu-controls, as expected (data not shown). Similar responses were observed in CREM*−/−* mice when basal lung function was measured without stimulation of AHR (supplementary Tab. S1a). However, there were no differences between OVA-treated knockout and wt animals.

**Figure 3:**
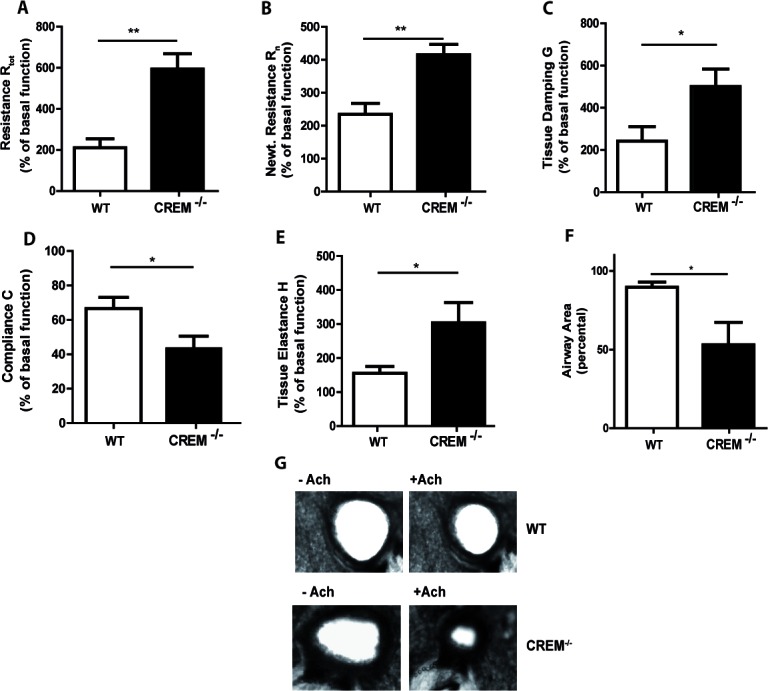
Genetic deletion of CREM enhances airway hyperresponsiveness after OVA-sensitization CREM^−/−^ and wt (C57Bl/6*129/SV) mice were sensitized and challenged with OVA and lung function was measured using the flexiVent system. **a.**-**e.** Maximal changes of lung function parameters (total lung resistance R_tot_, central airway resistance R_n_ (Newtonian resistance), tissue resistance G, compliance C and tissue elastance H) upon Ach stimulation (1mg, the highest non-lethal dose in C57Bl/6*129/SV). Basal lung function without sensitization and OVA-challenge was used as an internal standard (100%) for each mouse. **f.** Airway area of PCLS of wt and CREM^−/−^ mice after stimulation with 10μM Ach. Values were normalized to the airway area of unstimulated slices. **g.** Exemplary PCLS of wt and CREM^−/−^ mice non-stimulated and stimulated with Ach with a concentration of 10μM Ach. **a.**-**f.** Results represent means ± SEM with *n* = 6 in each group; *p < 0.05, **p < 0.01. See also supplementary Figure S3.

**Figure 4:**
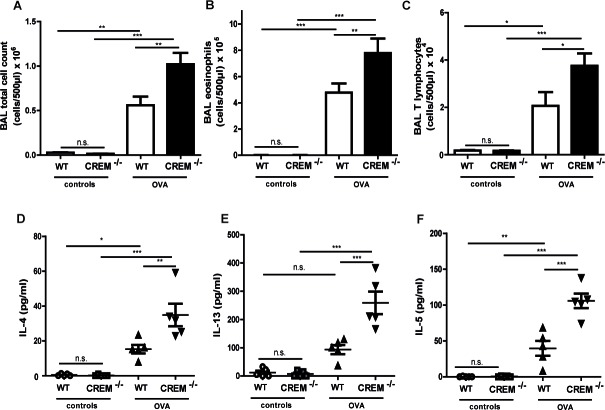
Genetic deletion of CREM enhances typical cell influx and production of T_H_2 cytokines *in vivo* after OVA-sensitization Allergic response of CREM^−/−^ and wt (C57Bl/6*129/SV) animals was measured after OVA-sensitization and OVA-challenges. Counts of total cells **a.**, eosinophils **b.** and T lymphocytes **c.** and BAL fluid levels of IL-4 **d.**, IL-13 **e.** and IL-5 **f.** in sensitized/challenged and control animals. **a.**-**f.** Results represent means ± SEM with *n* = 5 in all groups.*p < 0.05, **p < 0.01, ***p < 0.001.

In contrast, CREM deficiency led to strong AHR towards nebulized Ach compared to sensitized wt mice (Fig. 3a-3e, supplementary Fig. S4a-e and supplementary Tab. S1b). The maximal total lung resistance (R_tot_) was approximately 3-fold higher in sensitized CREM^−/−^ mice compared to sensitized wt animals (Fig. 3a) (1mg, the highest non-lethal Ach dose in C57Bl/6*129/SV wt mice). This increase is in accordance with the effects observed in both central and peripheral airways (R_n_/G) (Fig. 3b-3c). Total lung compliance (C), an indicator for lung elasticity, was reduced (Fig. 3d). Accordingly, the aggravated tissue elastance (H) was nearly doubled in sensitized knockout animals (Fig.3e). Non-sensitized animals showed no significant difference in AHR between both groups (data not shown). To confirm the AHR increase in sensitized CREM^−/−^ animals, we analyzed airway responses ex vivo using precision cut lung slices (PCLS). We could see that airways from sensitized wt animals showed only moderate bronchoconstriction in response to 10μM Ach while the airway area in sensitized CREM^−/−^ animals was nearly halved (Fig. 3f-3g). Alu-controls exhibited no differences between wt and CREM^−/−^ animals in the PCLS samples (supplementary Fig. S4f). When BAL fluid was analyzed, we could see that in control animals infiltrated cells were nearly absent, whereas OVA-sensitization and challenge led to the typical cell infiltration mainly comprising eosinophils and T cells into the alveolar space (Fig. 4a-4c). Total cell numbers in BAL fluid were doubled in sensitized CREM^−/−^ mice compared to wt animals (Fig. 4a). This strong difference was mainly due to the eosinophils (Fig. 4b) and T lymphocytes (Fig. 4c). Similar findings were made with respect to the T_H_2 cytokines IL-4, IL-5 and IL-13 that were present in the BAL fluid of OVA-treated mice and more than doubled in CREM^−/−^ mice (Fig. 4d-4f). The amount of recovered BAL did not differ significantly between the wt and CREM^−/−^ groups (data not shown). No significant differences were detected regarding the IgE-levels of wt animals compared to CREM^−/−^ mice (supplementary Fig. S4g). Additionally, *IL17* mRNA levels did not differ between both groups in total lung tissue (supplementary Figure S4h).

### Transgenic overexpression of CREMα in T cells diminishes T_H_2 type cytokines *in vitro*

**Figure 5:**
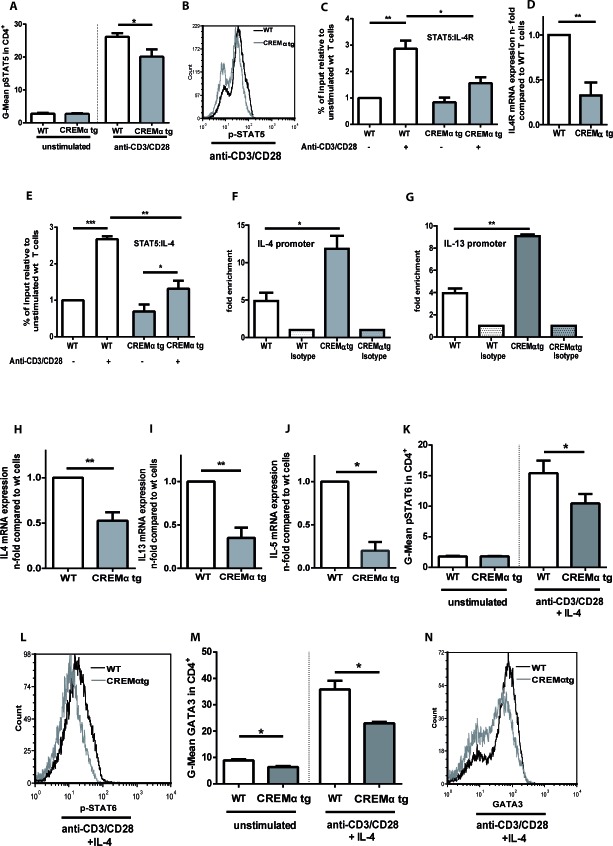
Transgenic overexpression of CREM decreases production of T_H_2 type cytokines in a pSTAT5 dependent manner **a.** CD4^+^ T cells from CD2CREMαtg and wt (FVB) mice were stimulated with plate-bound anti-CD3 and anti-CD28 for 24 hours and pSTAT5 was measured by flow cytometry. Mean fluorescence intensity (MFI) values for pSTAT5 were determined. Bars show the means of 6 experiments ± SEM. **b.** Representative histograms of pSTAT5expression. **c.**-**e.** Isolated T cells from CD2CREMαtg and wt mice were stimulated as in **a.** ChIP assay was performed with anti-STAT5 or control rabbit polyclonal IgG. Binding of the transcription factor STAT5 to the HSII site of the *Il-4* gene **c.** and to the *Il-4 receptor α* promoter site GAS3 **e.** was compared in wt and CREMαtg mice. Results represent means ± SEM with *n* = 4 experiments. **d.** RNA was extracted from T cells from CD2CREMαtg and wt mice and qRT-PCR was performed with primers specific for *Il4receptor α*. Bars show n-fold levels of *Il4R* expression in CD2CREMαtg T cells compared to wt T cells (set as 1). **f.**-**g.** ChIP assay was performed with anti-CREM or control rabbit polyclonal IgG. Binding of the transcription factor CREM to the HSII site of the *Il-4* gene (f) and to the *Il-13* promoter site (g) was compared in wt and CREMαtg mice. Results represent means ± SEM with *n* = 4 experiments. (h-n) CD4^+^ T cells from CREM^−/−^ and wt mice were stimulated in the absence or presence of IL-4. RNA was isolated and qRT-PCR was performed with primers specific for *Il4, Il5* and *Il13* **h.**-**j.**Graphs show fold-change compared to T_H_0 cells, which were stimulated with plate-bound anti-CD3 and anti-CD28 in the absence of IL-4. Flow cytometric analyses were performed for pSTAT6 **k.**-**l.** and GATA3 **m.**-**n.** after 3-5 days. Results represent means ± SEM with *n* = 3 experiments; **a.**-**n.** *p < 0.05, **p < 0.01, ***p < 0.001. See also supplementary Figure S5.

We demonstrated that genetic deletion of CREM enhances the T_H_2 response *in vitro* and *in vivo*. To determine whether CREM overexpression results in the opposite effect, we used a mouse which selectively overexpresses CREMα in T cells under the control of the CD2 promoter and that is characterized by decreased IL-2 expression [30]. This mouse showed previously enhanced disease severity in a lpr^−/−^ autoimmunity model [30]. In accordance with the data obtained from the CREM^−/−^ mice, CD2CREMαtg T cells presented the opposite phenotype with reduced IL-2 dependent pSTAT5 expression (Fig. 5a and 5b). As a consequence, pSTAT5 binding to the *Il-4r* promoter was significantly decreased (Fig. 5c) in transgenic mice. This correlated with reduced *Il4r* mRNA levels, known to be dependent on IL-2 (Fig. 5d). In addition, the binding of pSTAT5 to the *Il-4* promoter was decreased (Fig. 5e), while CREM binding to the *Il-4* and the *Il-13* promoter was strongly enhanced in the CD2CREMαtg T cells (Fig. 5f-5g, supplementary Figure S5a-b). As a consequence, *Il4* as well as *Il13* mRNA expression was decreased under T_H_2 conditions, this was true for *Il5* mRNA as well. (Fig. 5h-5j). In addition, pSTAT6 and GATA3 protein expression measured by flow cytometry exhibited consistent significant differences in T_H_2 polarizing conditions (Fig. 5k-5n). Thus, transgenic overexpression of CREMα in T cells clearly reduces transcription of T_H_2 pathways and nicely corroborates the phenotype of CREM^−/−^ T cells.

### Transgenic overexpression of CREM in T cells ameliorates the asthmatic response *in vivo*

**Figure 6:**
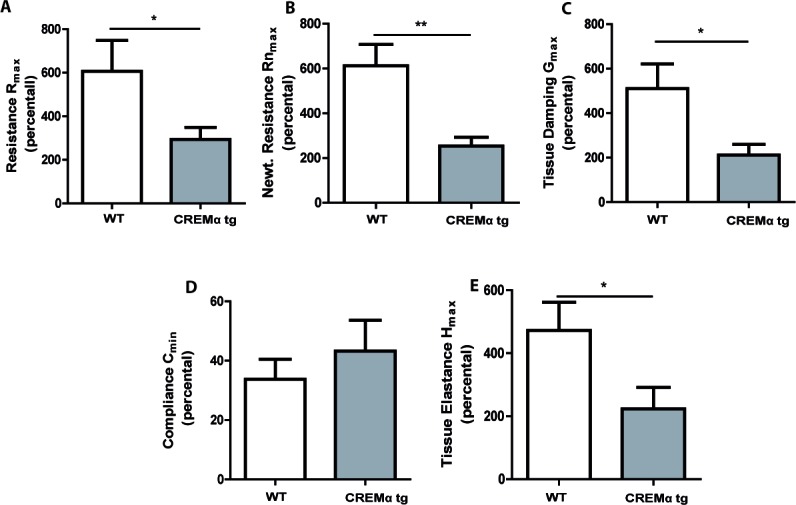
Transgenic overexpression of CREM decreases airway hyperresponsiveness in OVA-sensitized mice Airway hyperresponsiveness upon Ach stimulation (0.1mg, the highest non-lethal Ach dose in FVB wt mice) of OVA-sensitized and challenged CD2CREMαtg and wt (FVB) mice was measured using the flexiVent system. Total lung resistance R_tot_ **a.**, central airway resistance R_n_ (Newtonian resistance) **b.** and tissue resistance G **c.**, compliance C **d.** and tissue elastance H **e. a.**-**e.** Basal lung function was used as internal standard (100%) for each mouse. Results represent means ± SEM with *n* = 6 in both groups; *p < 0.05, **p < 0.01. See also supplementary Figure S6.

**Figure 7:**
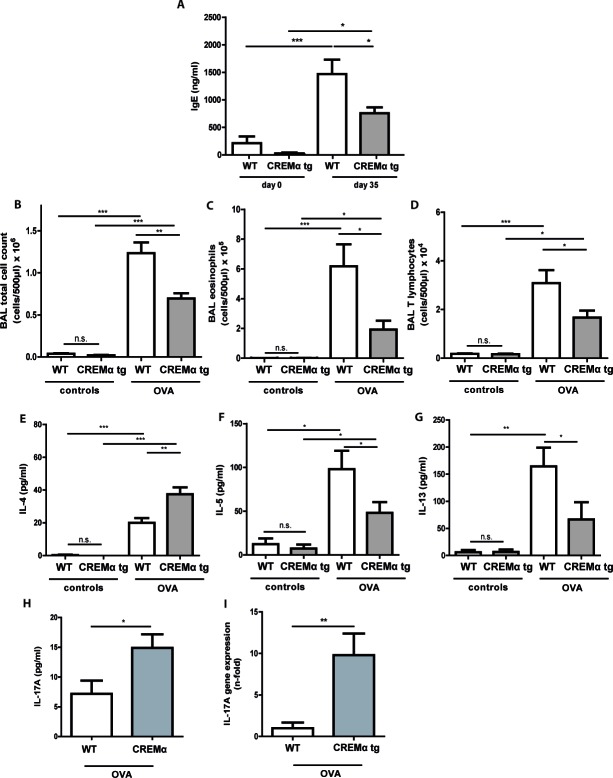
Transgenic overexpression of CREM decreases cell influx and production of T_H_2 type cytokines *in vivo* in OVA-sensitized mice Allergic responses of CD2CREMαtg and wt (FVB) animals were measured in the lung after OVA-sensitization. Total IgE serum levels **a.** of OVA-treated animals and controls. Counts of total cells **b.**, eosinophils **c.** and T lymphocytes **d.** and BAL fluid levels of IL-4 **e.**, IL-5 **f.**, IL-13 **g.** and IL-17 **h.** as well as *Il17* mRNA **i.** in sensitized and control animals. **a.**-**i.** Results represent means ± SEM with *n* = 5 in all groups. *p < 0.05, **p < 0.01, ***p < 0.001.

We further explored whether our CD2CREMαtg mice would be protected from asthma using the same asthma model. CREMα overexpression in T cells only slightly altered the basal lung functions after sensitization (supplementary Tab. S2a). In contrast to the baseline measurements, AHR was clearly reduced in CD2CREMαtg mice compared to wt (Fig. 6a-6e). Consequently, we could find a dose-dependent increase in airway resistance upon Ach provocation in wt and CD2CREMαtg mice, and a significantly stronger increase was observed in sensitized wt compared to transgenic animals. Indeed, upon Ach stimulation (0.1mg, the highest non-lethal Ach dose in FVB wt mice), total lung resistance (R_tot_) was halved in CD2CREMαtg mice compared to OVA-treated wt mice (Fig. 6a, supplementary Fig. S6a-e and supplementary Tab. S2b). This reduction is explained by the effects measured in central and peripheral airways (R_n_/G) (Fig. 6b-6c). Total lung compliance C (Fig. 6d) was insignificantly elevated in transgenic animals but the tissue elastance (H) was approximately halved in CD2CREMαtg compared to wt animals (Fig. 6e). Total IgE levels were halved in sensitized CD2CREMαtg mice in comparison to OVA-treated wt animals (Fig. 7a). Leukocytes were nearly absent in BAL fluid of non-sensitized control animals (Fig. 7b-7d). However, OVA-sensitization resulted in increased total cell numbers largely due to the pronounced invasion of eosinophils and T cells (Fig. 7b-7d). CD2CREMαtg mice demonstrated strikingly reduced numbers of all cells in BAL fluid. Accordingly, IL-5 and IL-13 were downregulated in OVA-sensitized transgenic mice, but surprisingly, and in contrast to the in vitro data, IL-4 levels in BAL were enhanced (Fig. 7e-7g). Strikingly, IL-17A levels in BAL and *Il17* mRNA levels of total lung homogenate were also higher in sensitized and challenged CD2CREMαtg mice than in wild type mice (Fig 7h-7i), pointing towards a stronger effect of CREM on IL-2 expression and T_H_2 differentiation compared to IL-17 expression in the outcome of ovalbumin-induced asthma.

## DISCUSSION

Here we provide evidence that CREM is a critical regulator of asthmatic inflammation by influencing the T_H_2 response *in vitro* and *in vivo*. CREM expression is downregulated in T cells derived from children with recurrent wheeze and in adults with history of atopy. Apparently, CREM levels are of functional importance, since CREM^−/−^ mice display increased inflammation and disease severity in an experimental asthma model. Moreover, CREM^−/−^ T cells express significantly enhanced levels of T_H_2-cytokines IL-4, IL-5 and IL-13, and of the lineage transcription factor GATA3. We suggest that this effect is partially mediated through a CREM-dependent IL-2 regulation, since pSTAT5 and IL-4R are upregulated and the blockade of IL-2 suppresses the expression of T_H_2 type cytokines in CREM^−/−^ T cells. Vice versa, transgenic overexpression of CREMα selectively in T cells results in reduced IL-2 expression [29], decreased pSTAT5 and IL4R levels and diminished T_H_2 type cytokines *in vitro* and in an asthma model *in vivo*. In addition a direct binding of CREM to the *Il-4* and *Il-13* locus suppresses transcription of both cytokines.

CREM belongs to the family of ATF/CREB type bZip transcription factors, members of which are already associated with T_H_2 differentiation processes. CREB itself has been involved in the T_H_2 differentiation by binding and suppression of the IFN-γ promoter during T_H_2 differentiation [32]. In addition, ATF3 directly binds to CRE sites in the IL-4, IL-5 and IL-13 promoter regions and ATF3 binding sites were found enriched in a recent epigenomic analysis of regulated genes that play a role for T_H_2 memory cell differentiation and asthma susceptibility [28]. In our study, using the same primers as in [27] we also show enhanced binding of CREM to the *Il-4* and *Il-13* promoter CRE sites suggesting a direct repressive effect of CREM on *Il4* transcription. In the CREM transgenic mice this also results in decreased total IgE-levels, while this is not the case in the CREM^−/−^ mice pointing to a more complex regulation and a possible effect of CREM also in B cells.

Until now the pathophysiological role of CREMα has been shown in the context of autoimmunity. Patients with SLE display severely altered T lymphocytic signaling pathways due to disease-related CREM overexpression [20, 33]. Hereby, enhanced expression of CREMα suppresses amongst others the expression of IL-2, TCRζ-chain and c-fos by direct inhibition of promoter activity [34, 35]. Influencing CREM function or CREM mediated pathways are potential therapeutic targets. Inhibition of CamKIV (calcium and calmodulin dependent kinase IV), which decreases CREM activation, was beneficial in murine models of SLE [36]. Furthermore treatment with IL-2 was successful in refractory human SLE [37] and we and others have shown that it delays autoimmunity in murine lupus models as well [38, 39]

With regard to asthma and allergy, IL-2 appears to exert harmful effects as IL-2 induced STAT5 phosphorylation drives T_H_2 differentiation [16]. In line with this Interleukin-2 inhalation therapy temporarily induces asthma-like airway inflammation [40] and daclizumab, a monoclonal antibody directed against CD25 subunit of IL2-R, improves asthma control in patients with moderate to severe persistent asthma [41]. CREM^−/−^ mice express enhanced IL-2 levels, which in addition to above mentioned direct binding of CREM results in activation of critical regulatory elements from the T_H_2 cytokine locus. We could show that CREM deficiency promotes IL-2 and pSTAT5-mediated T_H_2 responses including enhanced expression of IL4R, which primes cells for responsiveness to IL-4 and subsequently leads to enhanced STAT6-dependent GATA3 induction. We could also demonstrate the opposite effect that CREM overexpression in T cells downregulates IL-2 and subsequently STAT5- and STAT6 mediated responses.

We suggest an upregulation of CREM in T cells represents a physiologic feedback loop. We have shown before that CREM is induced during T cell activation [34], binds to the IL-2 promoter and restricts accessibility of other transcription factors by mechanisms including recruitment of histone deacetylase 1 (HDAC1) [42]. Thus, CREM-mediated repression of IL-2 as well as direct binding of CREM to the T_H_2 locus prevents excessive T_H_2 differentiation. The factors and mechanisms involved in the inhibition of CREM expression in T cells during allergic sensitization will be a further topic of our future investigation since this could have therapeutic implications for allergic diseases. We have shown before that CREM can be induced in antigen-presenting cells by TLR 4, 7 and 9 agonists [43]. This could also represent an additional therapeutic mechanism for adjuvant treatment in allergen specific immunotherapy. Rauen *et al*. demonstrated that CREMα is not only a transcriptional repressor, but it also directly binds to a CRE region within the *IL-17A* promoter in human T cells and increases its activity by histone and DNA modifications [44]. Apparently, we were able to confirm this finding also for the murine *Il-17a* promoter using the CD2CREMαtg mice, representing a central part of this project [30]. These mice are otherwise healthy and show no obvious T cell phenotype in homeostatic conditions including expression of regulatory T cells [30]. However in inflammatory conditions like in contact dermatitis [30] or in the ovalbumin-asthma model these mice are characterized by enhanced expression of *Il17*. Nevertheless, the enhanced expression of *Il17* in the lungs of CD2CREMαtg mice does not result in a more severe asthma phenotype. This is interesting since IL-17 has been considered to be a central cytokine in asthma pathogenesis [45–47]. On the other hand, Schnyder-Candrian et. al have shown, that IL-17 has a dual role in asthma and that exogenous IL-17 supplemented in the effector phase ameliorates the asthmatic response [48].

Our findings demonstrated that the overexpression of a single transcription factor in T cells ameliorates the T_H_2 response and, as a functional consequence, suppresses the asthmatic inflammation in a murine model. We recently showed that in opposite, CREM overexpression in T cells aggravates an LPS-mediated model of acute lung injury, while CREM^−/−^ T cells mediate protection [49]. Importantly, the overexpression of CREMα in lymphocytes is a hallmark of patients with SLE that are also protected against allergic diseases [50] but are more susceptible to complications from sepsis, including those with gram-negative bacteria [51]. We suggest that these findings could potentially be explained by the ability of CREMα to suppress IL-2 and T_H_2 type cytokines.

In summary, these novel insights highlight a possible role of CREM and CREM-dependent functional T cell alterations in T_H_2 lineage decisions. Influencing the levels of CREM could open new ways for the treatment of allergic diseases.

## EXPERIMENTAL PROCEDURES

A full description of methods is presented in the Experimental Procedures’ section in this article's Online Repository at http://www.oncotarget.com/supplemental-information

### Study cohort of children for the gene expression analyses of CREM in CD4^+^ T-cells

The patients were 6-yr-old wheezers and matched healthy controls belonging to a microarray analysis study of Kapitein et al.[18]. RNA samples of unstimulated peripheral CD4^+^ T-cells were used for new cDNA synthesis (*n* = 7 healthy controls and *n* = 10 wheezers). CREM gene expression analyses were conducted using human primers (see Supplementary Tab. S3). The gene expression profile was calculated as ΔCT values compared to the housekeeping gene GAPDH.

### Adult study cohort for the gene expression analyses of CREM in PBMC

As part of the expression analysis CohorT (EXACT) in 2012, adult subjects from German descent (*n =* 61, 28 males) were recruited, from whom 50 mL peripheral blood were obtained for isolation of peripheral blood mononuclear cells (PMBC), DNA and RNA. Unstimulated PBMCs were used as baseline control, while stimulated were exposed for 48 h to phytohemagglutinin (PHA), *D.pteronyssinus (*Derp1) and peptidoglycane (Ppg). Subsequently RNA was extracted followed by cDNA synthesis using 1 μg total RNA. Gene expression analyses for *CREM* were then conducted in 43 adults; 26 “controls” and 17 “allergic” using Taqman probes and 18sRNA as housekeeping gene.

### Animals

Experiments were performed with 8 to 12 weeks old male C57Bl/6*129/SV- and FVB-wild type (wt) mice, CREM^−/−^(C57Bl/6*129/SV) (29) and CD2CREMαtg-mice (FVB) [30]. The study was approved by the regional governmental authorities and animal procedures were performed according to the German animal protection law.

### Experimental design

On day 0, 14 and 21 CREM^−/−^, CD2CREMαtg and wt C57Bl/6*129/SV, and FVB mice were sensitized with ovalbumin (OVA) and aluminumhydroxide (Alu). On day 28 and 29, sensitized animals were exposed to nebulized OVA (1%) for 30 min each day, whereas controls were exposed to NaCl (0.9%). On day 35, mice were mechanically ventilated with the flexiVent ventilator (SCIREQ, Canada) and lung functions were measured by the forced oscillation technique. AHR was provocated with rising acetylcholine (Ach) aerosol concentrations.

### T cell isolation and T cell differentiation

Murine T cells were isolated from splenocytes using the MACS protocol (Miltenyi, Germany). CD4^+^ T cells (2×10^6^ per mL) were then incubated with plate-coated anti-CD3 (10 μg/mL) and anti-CD28. For T_H_2 priming cells were cultured in the presence of 30 ng/mL IL-4. To neutralize IL-2 signaling cells were cultured in the presence of 2μg/mL anti-IL-2 antibody. After 48 h T cells were harvested for RNA extraction. Cell culture supernatants were utilized for ELISA measurements of IL-5 and cells were analyzed by flow cytometry after 4-5 days.

### Chromatin-immunoprecipitation (ChIP)

ChIP was performed as described in Supplementary Methods. DNA was recovered and subjected to quantitative PCR analysis. Primer sequences used for quantitative real-time PCR were for STAT5 binding site HSII of the *Il-4* promoter adapted from Zhu et al. [15]; and for STAT5 *Il-4 receptor α* promoter binding site GAS3 adapted from Liao et al. [16] (see Supplementary Tab. S4). The immunoprecipitated DNA was calculated as relative to the respective input DNA and the percentage of input was compared relative to the unstimulated wt cells.

### RNA isolation and real-time PCR

Total RNA from cells was isolated using RNeasy Mini Kit (Qiagen, Germany). cDNA was then generated using First Strand cDNA Synthesis Kit (Fermentas, Germany) according to the manufacturer's instructions. Standard real-time PCR was carried out on TaqMan 7900 (Applied Biosystems by Life Technologies, Germany) using the DNA intercalating dye SYBR Green. Primer sequences can be found in Supplementary Tab. S5. Relative quantification method as described in [52] was applied and Delta C_t_ (ΔC_t_) values were determined.

### Precision-cut lung slices

Precision-cut lung slices (PCLS) were prepared according to Martin et al. [53]. Airway area was calculated with Optimas 6.5 (Media Cybernetics, USA) to evaluate bronchoconstriction after Ach provocation.

### Statistical analyses

All statistical analyses and the subsequent graphics generation were performed using GraphPad Prism version 6.0 and JMP 7.0.1. Mixed model analyses of lung mechanics were performed using the SAS 9.1 software. A *p*-value <0.05 was considered significant.

## SUPPLEMENTARY MATERIAL FIGURES AND TABLES


