# Supplementary material for: The cAMP response element modulator (CREM) regulates TH2 mediated inflammation
Source: Oncotarget. 2015 Oct 8;6(36):38538–51. doi: 10.18632/oncotarget.6041 (PMC4770719; doi:10.18632/oncotarget.6041)
Supplement: Supplementary file 1 [file oncotarget-06-38538-s001.pdf]

The cAMP response element modulator (CREM) regulates $T_{H}2$ mediated inflammation

Supplementary Material

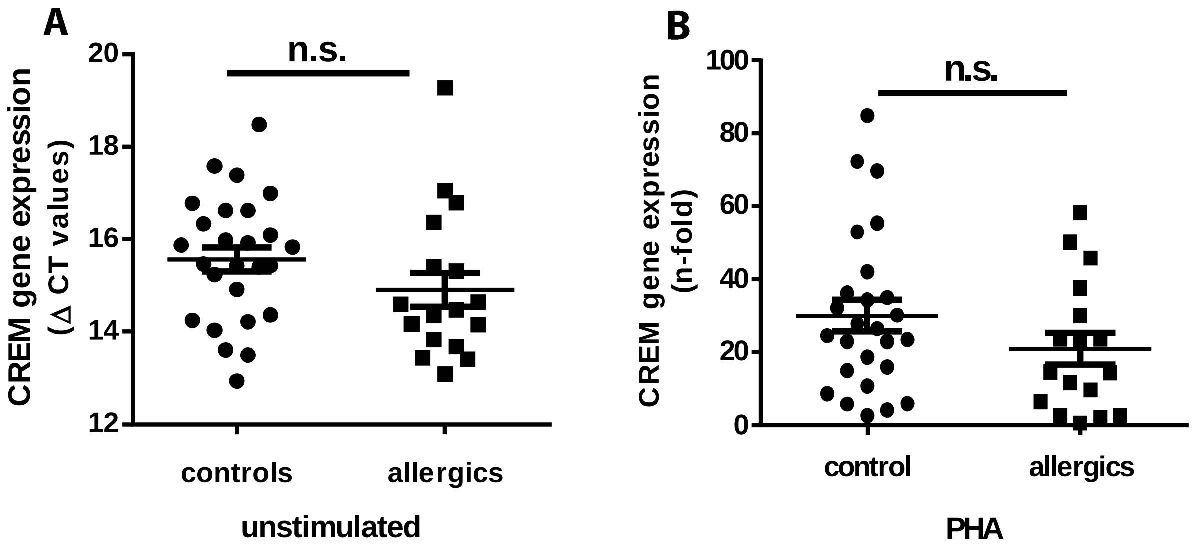

C

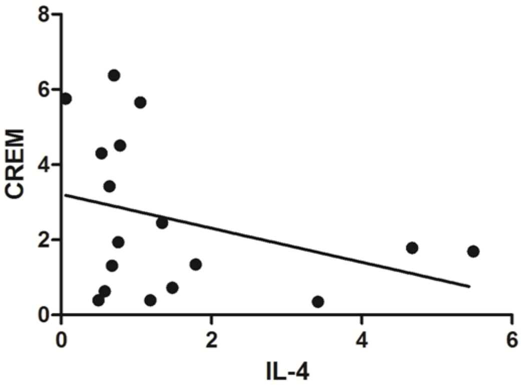

D

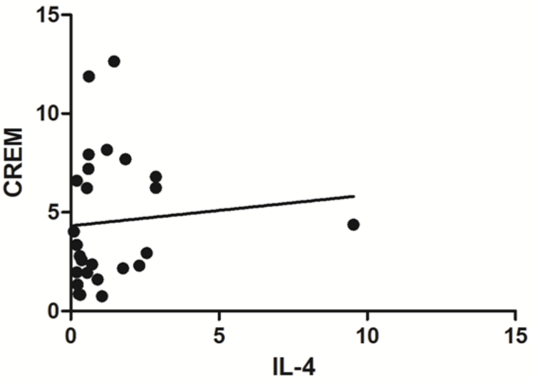

Figure S1:

PBMCS from adults with allergies (n=17) and healthy controls (n=26) were isolated and CREM-levels were analyzed by qRT-PCR. Results represent Δ-CT values compared to the housekeeping gene 18srRNA **(a)**. Additional isolated cells were stimulated for 48h with PHA, RNA was extracted and CREM levels were analyzed by qRT-PCR. Results show n-fold CREM expression normalized to unstimulated PBMC **(b)**. All results represent means ± SEM.

(c) Correlation of fold change of CREM and IL-4 in allergics after stimulation of PBMCs with Derp1 (R-0.3377, n.s.). **(d)** Correlation of fold change of CREM and IL-4 in controls after stimulation of PBMCs with Derp1 (R=0.08, n.s.).

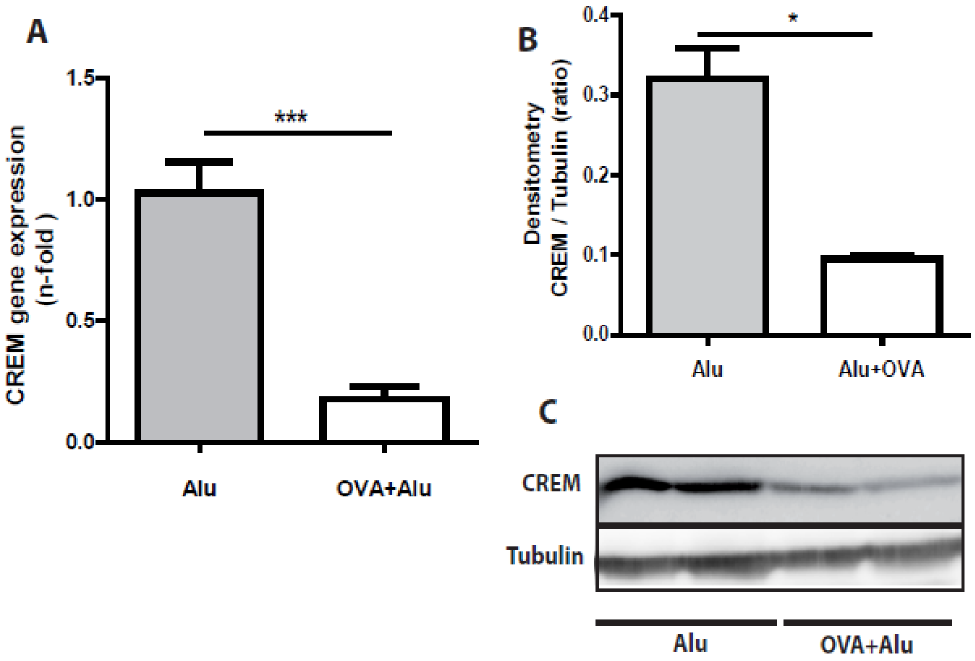

Figure S2:

(**a-c**) Wt C57Bl/6*129/SV mice were sensitized with OVA and alu or with alu only. Levels of CREM gene expression in lung tissue were analyzed by qRT-PCR using CREM-specific primers; n=5 in both groups **(a).** Protein of lung tissue was isolated, subjected to western blot analyses and stained with anti-CREM or tubulin as loading control; n=3 in both groups **(b-c).**

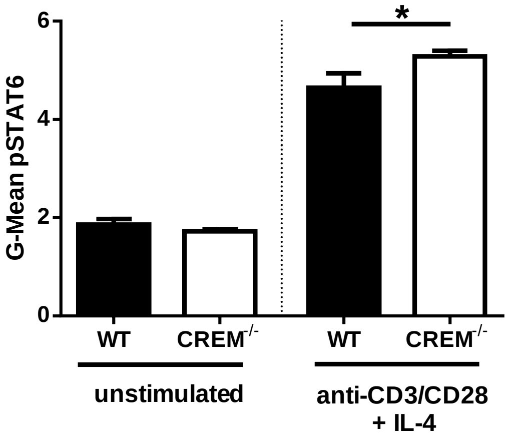

Figure S3:

CD4+ T cells from CREM-/- and wt mice were stimulated with plate-bound anti-CD3 and anti-CD28 in the presence of IL-4. pSTAT6 levels were determined by flow cytometry. n=3 in both groups.

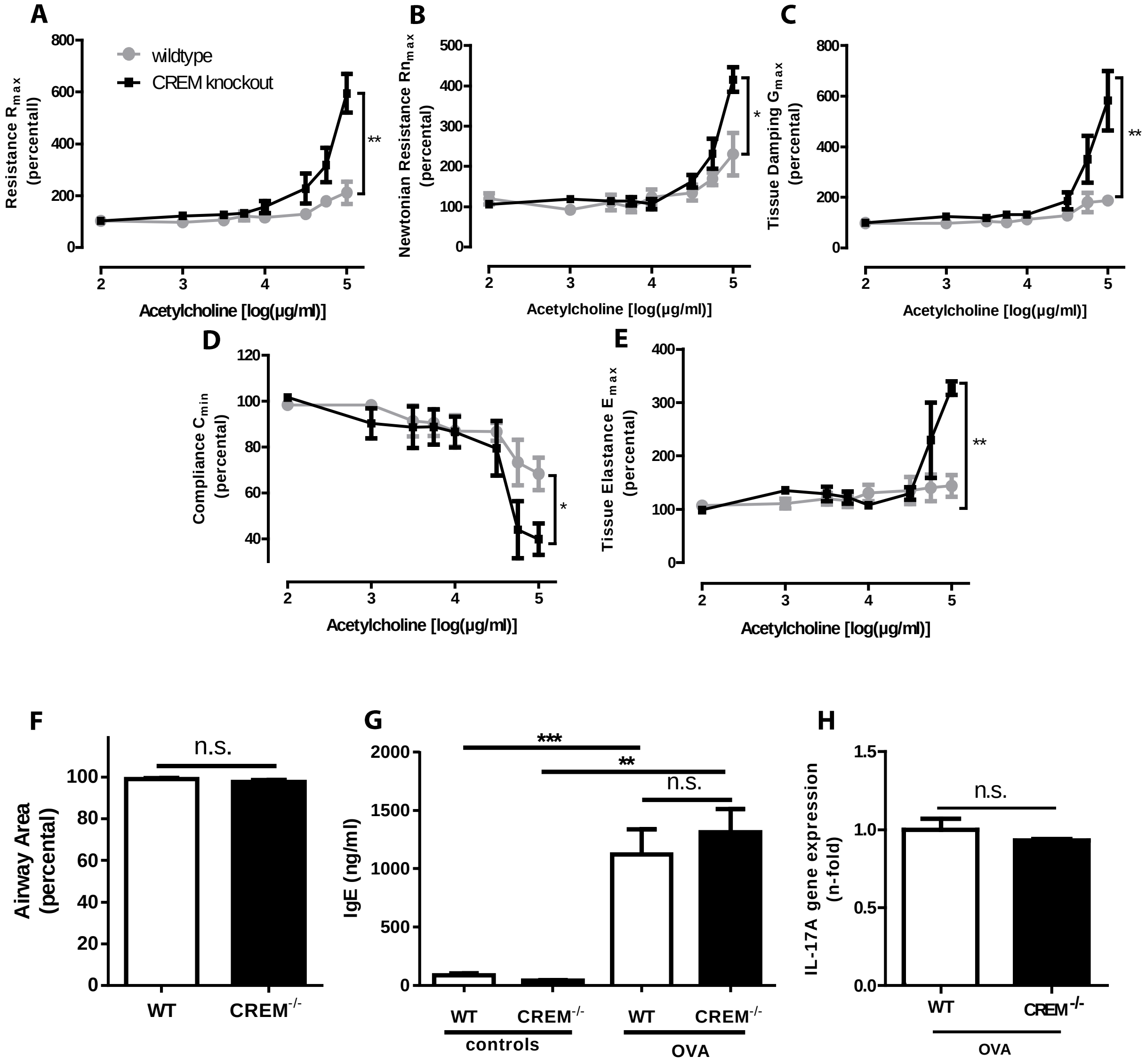

Figure S4:

(a-e) Airway hyperresponsiveness of sensitized and OVA-challenged CREM$^{-/-}$ and wt (C57Bl/6*129/SV) mice. Exemplary dose-response curves to nebulized Ach of total lung resistance R_{tot} **(a)**, central airway resistance R_n (Newtonian resistance) **(b)** and tissue resistance G **(c)** as well as lung elastance E **(d)** and tissue elastance H **(e)**. n= 6 mice in each group. **(f)** Airway area of PCLS of non-sensitized wt and CREM$^{-/-}$ mice after stimulation with 10 µM Ach (n=3). Values were normalized to the airway area of unstimulated slices. **(g)** IgE-levels of wt and CREM$^{-/-}$ mice with and without OVA-sensitization. n=5 in non-sensitized

groups and n=7 in sensitized groups. All results represent means ± SEM. **(h)** mRNA levels of IL-17 in total lung homogenates of $CREM^{-/-}$ and wt (C57Bl/6*129/SV) mice were determined by qRT-PCR. n=5 in each group. All results represent means ± SEM.

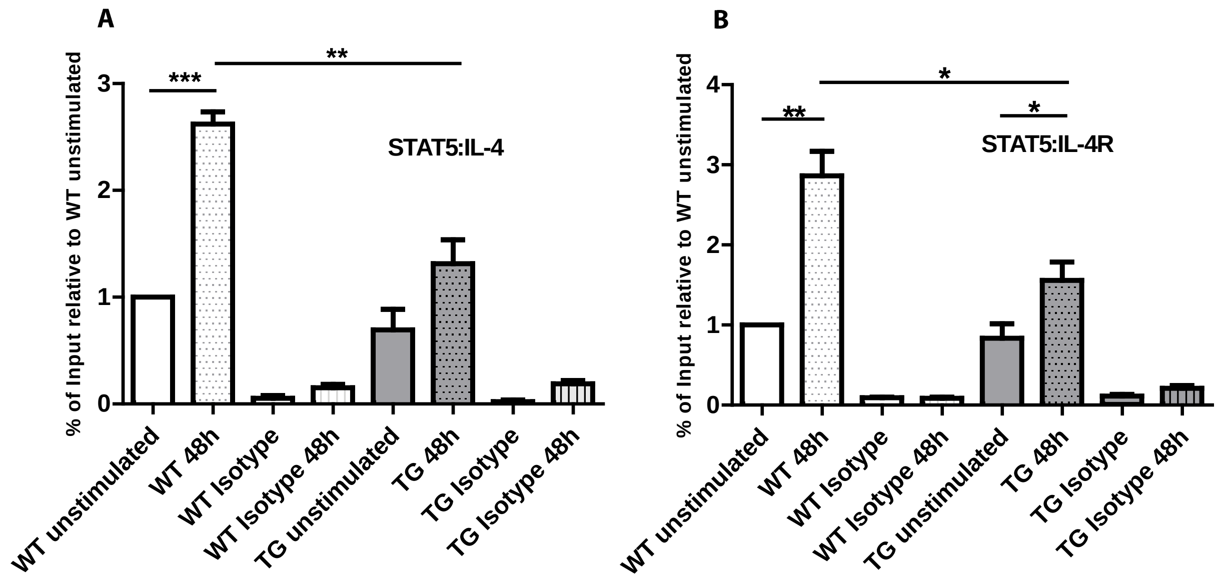

Figure S5:

(a-b), ChIP assay was performed with anti-STAT5 or control rabbit polyclonal IgG. Binding of the transcription factor STAT5 to the HSII site of the **(a)**, Il-4 promoter **(b)**, and to the Il-4R promoter site was compared in wt and CREMαtg mice. Results represent means ± SEM with n=4 experiments.

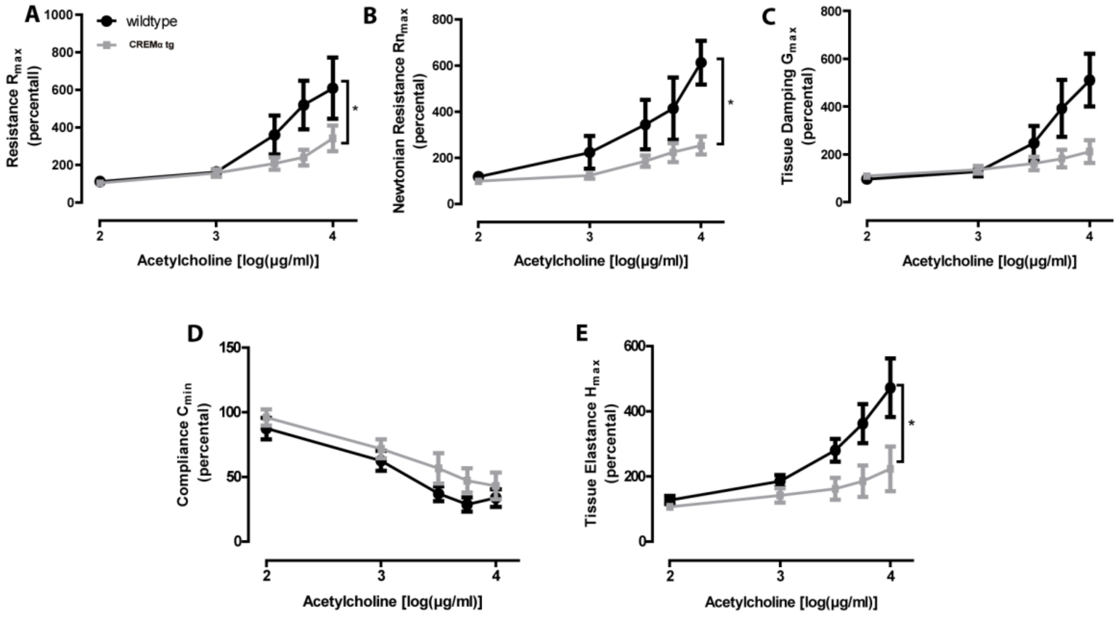

Figure S6:

(a-e) Airway hyperresponsiveness of sensitized CD2CREMαtg and wt (FVB) mice. Exemplary dose-response curves to nebulized Ach of total lung resistance R_{tot} **(a)**, central airway resistance R_n (Newtonian resistance) **(b)** and tissue resistance G **(c)** as well as lung elastance E **(d)** and tissue elastance H **(e)**. n= 6 mice in each group.

Supplementary Tables:

Table S1a: Basal lung function of non-sensitized and sensitized and OVA-challenged CREM$^{-/-}$ (KO) and wild type (C57Bl/6*129/SV) animals.

CREM-mice	wt control	KO control	wt OVA	KO OVA
Total Lung Resistance (R_{tot})	0.48 ± 0.02	0.65 ± 0.04	0.81 ± 0.09	0.88 ± 0.14
Compliance (C)	0.045 ± 0.003	0.036 ± 0.004	0.030 ± 0.002	0.031 ± 0.003
Newtonian Resistance (R_n)	0.30 ± 0.01	0.35 ± 0.01	0.51 ± 0.05	0.56 ± 0.09
Tissue Resistance (G)	2.63 ± 0.35	4.09 ± 0.41	4.99 ± 0.35	5.13 ± 0.33
TissueElastance (H)	24.1 ± 2.1	30.1 ± 4.0	35.2 ± 2.3	34.7 ± 3.3

On day 35 of the sensitization experiment mice were ventilated with the flexiVent setup and lung function parameters were measured. Total lung resistance (R_{tot}), total lung compliance (C), Newtonian (central airway) resistance (R_n), tissue resistance (G) and tissue elastance (H) (all in cmH_2Os/cm). Results represent means ± SEM (controls n=4 and OVA n=6 in both groups).

Table S1b: Lung mechanics of sensitized and OVA-challenged CREM$^{-/-}$ (KO) and wild type (C57Bl/6*129/SV) animals after nebulized Ach (1mg).

CREM-mice	wt OVA	KO OVA
Total Lung Resistance (R_{tot})	1.97 ± 0.59	4.89 ± 0.50
Compliance (C)	0.020 ± 0.0018	0.013 ± 0.0017
Newtonian Resistance (R_n)	1.09 ± 0.29	2.39 ± 0.53
Tissue Resistance (G)	13.02 ± 2.86	25.01 ± 4.06
TissueElastance (H)	52.52 ± 3.80	92.02 ± 21.12

On day 35 of the sensitization experiment mice were ventilated with the flexiVent setup and lung function parameters were measured after nebulized Ach. Total lung resistance (R_{tot}), total lung compliance (C), Newtonian (central airway) resistance (R_n), tissue resistance (G)

and tissue elastance (H) (all in cmH_2Os/cm). Results represent means ± SEM (n=6 in both groups).

Table S2a: <u>Basal lung function</u> of non-sensitized and sensitized and OVA-challenged CD2CREMαtg (CD2) and wild type (FVB) mice.

CREM-mice	wt control	CD2 control	wt OVA	CD2 OVA
Total Lung Resistance (R_{tot})	0.57 ± 0.02	0.58 ± 0.04	0.83 ± 0.08	0.64 ± 0.04
Compliance (C)	0.042 ± 0.001	0.045 ± 0.001	0.031 ± 0.002	0.041 ± 0.002
Newtonian Resistance (R_n)	0.35 ± 0.01	0.36 ± 0.03	0.43 ± 0.02	0.36 ± 0.02
Tissue Resistance (G)	3.65 ± 0.08	3.53 ± 0.19	5.30 ± 0.42	3.84 ± 0.11
TissueElastance (H)	24.8 ± 0.8	22.6 ± 0.8	30.1 ± 1.6	24.3 ± 1.1

Animals were ventilated with the flexiVent setup and lung function parameters were measured. Total lung resistance (R_{tot}), total lung compliance (C), Newtonian (central airway) resistance (R_n), tissue resistance (G) and tissue elastance (H) (all in cmH_2Os/cm). Results represent means ± SEM (controls n=8 and OVA n=6 in both groups).

Table S2b: Lung mechanics of sensitized and OVA-challenged CD2CREMαtg (CD2) and wild type (FVB) animals <u>after nebulized Ach (0.1mg)</u>.

CREM-mice	wt OVA	CD2 OVA
Total Lung Resistance (R_{tot})	5.69 ± 0.97	2.79 ± 0.34
Compliance (C)	0.0067 ± 0.0011	0.0097 ± 0.0014
Newtonian Resistance (R_n)	2.22 ± 0.42	0.83 ± 0.11
Tissue Resistance (G)	28.6 ± 7.24	10.59 ± 2.16
TissueElastance (H)	138.7 ± 15.62	62.27 ± 7.2

On day 35 of the sensitization experiment mice were ventilated with the flexiVent setup and lung function parameters were measured after nebulized Ach. Total lung resistance (R_{tot}),

total lung compliance (C), Newtonian (central airway) resistance (R_n), tissue resistance (G) and tissue elastance (H) (all in cmH_2Os/cm). Results represent means ± SEM (n=6 in both groups).

Table S3: Human qRT-PCR Primer

CREM	forward	5' -AGTCCCTGCCCTTTGTACACA-3'
	reverse	5’-GATCCGAGGGCCTCACTAAAC-3’
18S rRNA		5’-6-FAM-CGCCCGTCGCTACTACCGATTGG-3’-Tamra

Table S4: Murine ChIP Primer

STAT5 binding site of the *Il-4* promoter	forward	5'-AAACACATGACTTGGCTATGCTGTA-3'
	reverse	5'-AGGTCTTTGTTCCTGACCTCATGT-3'
STAT5 binding site of the *Il-4r* promoter	forward	5'-CCAGCCTGCGTTCAGTCTCT-3'
	reverse	5'-CACTGGTGGCAGCACTTAGAACT-3'

Table S5: Murine qRT-PCR Primer

β-Actin	forward	5’-GACTACCTCATGAAGATCCTCACC-3’
	reverse	5’-TCTCCTTAATGTCACGCACGATT-3’
Crem	forward	5’-AACTGTCCTCTGATGTGCCTGG-3’
	reverse	5’-TTGCCCCGTGCTAGTCTGATAT-3’
Il-2	forward	5’-GACCTCTGCGGCATGTTCTG-3’
	reverse	5’-TCATCGAATTGGCACTCAAATG-3’
Il-4	forward	5’-AGGTCACAGGAGAAGGGACGCC-3’
	reverse	5’-TGCGAAGCACCTTGGAAGCCC-3’
Il-5	forward	5’-GACATTCTCCTTGCAGTGTGAATGA-3’
	reverse	5’-GGGTAGTAAGGTTGTGGTATTTACTTTGC-3’
Il-13	forward	5’-GCCTGTTACACTCAAGGTGATGTG-3’
	reverse	5’-CTGTCACCATCTTTATTTCCGGTTT-3’
Il-4r	forward	5’-AGAGGGACCTGGCTTCTGAT-3’
	reverse	5’-CAAAAGGTGCCTGCACAAGG-3’

Supplemental Experimental Procedures:

Description of the adult study cohorts utilized for the gene expression analyses of *CREM*

As a part of the EXpression Analysis CohorT (EXACT), in 2012 adult subjects from German descent (*n*=61, 28 males) were recruited. The study protocol was approved by the Hannover Medical School ethic committee, Hannover, Germany and a written informed consent was acquired from each participant. 50 mL peripheral blood was obtained from the recruited individuals for the purposes of peripheral blood mononuclear cells (PMBC), DNA- and RNA-isolation. Unstimulated PBMC were used as a baseline control, while the stimulated were exposed for 48 h to innate and adaptive immune stimuli such as phytohemagglutinin (PHA), *D.pteronyssinus (*Derp1) and peptidoglycane (Ppg). Subsequently RNA was extracted (Macherey-Nagel GmbH & Co. KG, Dueren, Germany) from the cell pellets, followed by cDNA synthesis using as an input 1 µg total RNA (QuantiTect Reverse Transcription, Qiagen, Hilden, Germany).

Each participant filled out questionnaire providing information whether he suffered from one or more of the following immunologic conditions: asthma, hay fever, neurodermatitis (atopic eczema, endogenous eczema or atopic dermatitis), psoriasis, diabetes mellitus, rheumatoid disease, ulcerous colitis, Crohn’s disease, multiple sclerosis or any other autoimmune disorder. Participants also answered whether the respective condition(s) started during childhood or later in life and if it was still persisting at the time of their recruitment in the study. Additionally, information regarding participants’ smoking status (smokers or not) was obtained, as well as if they have ever had a skin prick test(s) conducted and what the results were.

Out of 57 adult individuals with a good quality RNA, derived from unstimulated and stimulated with all four stimuli PBMC, we excluded six subjects from our mRNA expression

analyses due to their reported asthma status and 8 patients with autoimmune diseases according to the questionnaire. Thus, gene expression analyses for *CREM* were then conducted in 43 adults, divided in two subgroups: “controls” and “allergic” individuals. As controls (*n*=26) we assigned those participants who have no illness reported, negative tests results or no tests ever conducted (since there was medical no reason to perform any tests). As allergics (*n*=17) we defined those who had a history of allergies, hay fever or neurodermitis and one or more of health conditions and/or had positive test results.

Peripheral blood mononuclear cells (PBMC) isolation and processing

Approximately 50 mL venous blood was drawn from each subject using Na-Heparin-Monovettevacutainers (SarstedtAG&Co, Nümbrecht, Germany). In a next step the blood was transferred into LeucoSep 50 mL tubes (Greiner Bio-One GmbH, Frickenhausen, Germany) prefilled with Ficoll-Paque PLUS (GE Healthcare Europe, Freiburg, Germany) and subjected to centrifugation gradient-based cell separation. The PBMC fraction was then washed with RPMI 1640 + Glutamax-I (Life technologies, Carlsbad, California, USA). The respective cell culture media contained: RPMI 1640 + Glutamax-I supplemented with heat-inactivated human serum (final concentration of 10%; Sigma-Aldrich Co., Steinheim, Germany), penicillin (200 U) and streptomycin (200 µg; Pen Strep, Life technologies, Carlsbad, California, USA). For each condition $5x10^6$ PBMC per plate well were seeded in 1 mL media. The following stimuli were then applied: phytohemagglutinin (PHA, to the final concentration of 5 µg/mL PHA; Sigma-Aldrich Co., Steinheim, Germany), *D. pteronyssinus*(Derp1; to the final concentration of 30 µg/mL; NA-DP1-2, Indoor Biotechnologies, Warminster, UK), and peptidoglycane (Ppg; to the final concentration of 10 µg/mL; Sigma-Aldrich, Germany). The stimulated cells were harvested after 48 hours, stored frozen at -80°C and subjected (at a later time point) to a total RNA isolation, as explained above. From each subject a fraction of

unstimulated PBMC was incubated for 15 min only in 1 mL cultivation media without any stimulation, then harvested and frozen at -80°C. Unstimulated PBMC were used to determine the basic mRNA expression levels of the targeted genes and to investigate the degree of inducibility upon allergen stimulation.

Animals:

Experiments were performed with 8 to 12 weeks old male C57Bl/6*129/SV- and FVB-wild type (wt) mice, $CREM^{-/-}$(C57Bl/6*129/SV)[1] and CD2CREMαtg-mice (FVB) [2]. The study was approved by the regional governmental authorities and animal procedures were performed according to the German animal protection law. All mice were bred in the animal facility of RWTH Aachen and were kept under specific pathogen-free conditions. Wt and transgenic mice were age-matched for all experiments.

Experimental design

On day 0, 14 and 21 $CREM^{-/-}$, CD2CREMαtg and wt C57Bl/6*129/SV, and FVB mice were injected i.p. with 230 µl ovalbumin and aluminumhydroxide (Alu) (OVA:10µg/Alu:1.5mg, Sigma, Germany). Control animals received only Alu. On day 28 and 29 sensitized animals were exposed to nebulized ovalbumin (1%) for 30min each day. Controls were exposed to NaCl (0.9%). On day 35, mice were tracheotomized with a 20-gauge cannula and connected to a ventilator device. All mice were mechanically ventilated with a tidal volume (Vt) of 10 mL/kg and a positive end-expiratory pressure (PEEP) of 2cmH_2O using the flexiVent (SCIREQ, Canada) ventilation setup. Breath rate was set as 150/min. Inspiratory fraction of oxygen was 0.21. Dynamic lung mechanics was measured by applying a sinusoidal standardized breath and analyzed with forced oscillation technique. We used a 1.2s, 2.5 Hz

single-frequency forced oscillation manoeuvre (SnapShot perturbation) and a 3s, broadband low frequency forced oscillation manoeuvre, containing 13 mutually prime frequencies between 1 and 20.5 Hz (Quick Prime perturbation). Total lung resistance (R_{tot}) and elastance (E) were calculated using *flexiVent*software (flexiWare 7.0.1, SCIREQ, Canada) by fitting measured SnapShot values to the linear single compartment model applying multiple linear regressions. Respiratory system input impedance was calculated from the QuickPrime data and Newtonian (central airway) resistance (R_n), tissue resistance (tissue damping) (G), and tissue elastance (H) were assessed by iteratively fitting the constant-phase model to input impedance. During the first 30min of ventilation time, baseline values were recorded using a standardized script with continuous measurements every 30s. Every 5min short volume controlled recruitment maneuvers (3s) were used to avoid atelectasis. AHR was provoked with increasing concentrations of nebulized acetylcholine (Ach).

Body temperature was rectally controlled and adjusted to 37±0.5°C during the entire ventilation period. Continuous data recording of heart rate and ECG was performed to monitor the function of the cardiovascular system.

Bronchoalveolar lavage(BAL)

Following ventilation, lungs were removed. The entire right lung was used for bronchoalveolar lavage (BAL) by instilling 700µl ice-cold PBS.

Cytokine measurements

Murine IL-4, IL-5, IL-13and total IgE were analyzed in supernatants of BAL or cell culture supernatants using sandwich ELISA kits according to manufacturer's protocols (Biolegend/eBioscience, Germany).

Flow cytometry and westernblot analyses

Absolute numbers of BAL cells and antibody-stained populations were measured by flow cytometric analysis with the FACS-Canto (BD Bioscience, Germany). A minimum of 10,000 counts were collected for evaluation. To analyze cell surface expression of CD4, CD3 and IL-4R, cells were washed and incubated with anti-CD3, anti-CD4 an anti-IL-4R antibodies (all from eBioscience, Germany) for 30 minutes. For intracellular measurements of STAT5 and STAT6 phosphorylation, cells were first fixed in 1.5% paraformaldehyde for 10 minutes and then permeabilized in ice-cold methanol for at least 10 minutes prior washing and incubation with anti–pSTAT5 or anti-pSTAT6 antibodies (BD Biosciences, USA).For measurement of intracellular IL-4 production, cells were treated with PMA, ionomycin and GolgiPlug (all from BD Biosience, USA) for 5 hours. To analyze intracellular cytokines and GATA3 expression, cells were fixed and permeabilized with FOXP3 staining buffer set (eBioscience, Germany) following the manufacturer's instructions and stained with anti-IL-4 and anti-GATA3 antibodies (eBioscience, Germany) for 30 min. Flow cytometry was carried out using FACSCanto II device (BD Biosciences, Germany).

To determine CREM protein levels in lung tissue of sensitized wt animals and controls, we performed CREM immunoblots with a anti-CREM antibody (Sc-440, Santa Cruz Biotechnology, USA). Tubulin was used as a loading control (SC-1616, Santa Cruz Biotechnology, USA). Immunoblots were scanned using a Fuji LAS-3000 Image Reader (Fuji

Photo Film, Japan). Densitometric analysis was performed with the AIDA Image Analyzer 2.0 (Raytest, Germany).

Chromatin-immunoprecipitation (ChIP)

3-4 million splenic T cells from CD2CREMαtg-mice (FVB) and FVB-wt mice were maintained in RPMI 1640 medium (Gibco, Germany) supplemented with 10% fetal calf serum (FCS) (Gibco, Germany) and 1% penicillin/streptomycin (Invitrogen, Germany). T cells were stimulated using plate-bound anti-CD3 and anti-CD28 antibodies (R&D, USA) or left unstimulated. Unstimulated T cells were immediately cross-linked with 1% formaldehyde (Merck, Germany)and stimulated T cells were cross-linked after 48 h. Cross-linking was terminated with 2M Glycine. The cells were then washed with cold phosphate-buffered saline (PBS) and lysed in buffer containing protease inhibitors (Complete MiniRoche, Switzerland) for 20 minutes on ice. Cell lysates were sonicated to shear DNA and sedimented. Supernatants were diluted in RIPA buffer. A proportion (10%) of the diluted supernatants was kept as input (PCR amplification of the total sample). For each immunoprecipitation reaction equal DNA amount was used. Samples were immunoprecipitated with 0.8 µg STAT5 (anti-STAT5 ChIP grade; Abcam, UK) antibody or its isotype control (rabbit polyclonal IgG; Abcam) at 4°C overnight. Protein G Dynabeads (Novex by Life technologies, Germany) were washed with RIPA buffer and incubated with the samples for 4 hours at 4°C. Protein-DNA-beads complexes were washed stringently with wash buffer and final wash buffer (X-ChIP protocol from Abcam). Protein-DNA complexes were eluted in 1% SDS, 100 mM $NaHCO_3$ for 15 minutes at 30 °C and reverse-crosslinked at 65°C overnight. Proteinase K (20 mg/mL) was added to the samples and incubated for 2 hours at 54 °C to eliminate disturbing proteins. DNA was recovered using the Wizard kit (Promega, Germany) and subjected to PCR analysis on an ABI prism 7300 real-time PCR system. Primer sequences used for real-time PCR were

for STAT5 binding site HSII (DNase I hypersensitivity site II) of the *Il-4*promoter adapted from Zhu et al.[3]; and for STAT5 *Il-4 receptor α* promoter binding site GAS3 (TTCN3GAA IFNγ activated sequence) adapted from Liao et al. [4] (see Supplementary Tab. 3). The immunoprecipitated DNA was calculated as relative to the respective input DNA, and the percentage of input was compared relative to the unstimulated wt cells.

Precision-cut lung slices

Precision-cut lung slices (PCLS) were prepared according to Martin *et al*. [5] in order to investigate airway reactivity upon Ach provocation. Airway area was calculated with Optimas 6.5 (Media Cybernetics, USA) to evAluate bronchoconstriction.

Statistical analyses

All data are presented as mean ± standard error (SEM). BoxCox transformation was performed to achieve homoscedasticity and normal distribution, when necessary. In the *in vivo* experiments and the human gene expression analyses, differences between two groups were evaluated using unpaired two-sided Student's t-test when data were normally distributed. Otherwise non-parametric Mann-Whitney test was performed. Comparisons with more than two groups were analyzed with One-way-ANOVA and corrected by FDR for multiple comparison. Non-parametric data were analyzed with the Kruskall-Wallis test followed by Dunn's post-hoc-test. Normalized Data (changes in mRNA expression) were compared using the one-sample t-test.

In vitro results with a concrete hypothesis were tested with one-sided Student's t-test when data were normally distributed. The results of experiments of one day of the *in vitro* T_H2-differentiations were paired for statistical analyses.

All statistical analyses and the subsequent graphics generation were performed using GraphPad Prism version 6.0 (GraphPad Software, USA) and JMP 7.0.1 (SAS Institute, USA). Mixed model analyses of lung mechanics were performed using the SAS 9.1 software (SAS Institute, USA). A p-value lower than 0.05 was considered to be significant. We used the Grubbs-test or ESD-Method to exclude outlier values from the measurement.

1. Blendy, J.A., K.H. Kaestner, G.F. Weinbauer, E. Nieschlag, G. Schutz, Severe impairment of spermatogenesis in mice lacking the CREM gene. Nature, 1996. 380(6570): 162-5.

2. Lippe, R., K. Ohl, G. Varga, T. Rauen, J.C. Crispin, Y.T. Juang, S. Kuerten, F. Tacke, M. Wolf, K. Roebrock, T. Vogl, E. Verjans, N. Honke, et al., CREMalpha overexpression decreases IL-2 production, induces a T(H)17 phenotype and accelerates autoimmunity. J Mol Cell Biol, 2012. 4(2): 121-3.
3. Zhu, J., J. Cote-Sierra, L. Guo, W.E. Paul, Stat5 activation plays a critical role in Th2 differentiation. Immunity, 2003. 19(5): 739-48.
4. Liao, W., D.E. Schones, J. Oh, Y. Cui, K. Cui, T.Y. Roh, K. Zhao, W.J. Leonard, Priming for T helper type 2 differentiation by interleukin 2-mediated induction of interleukin 4 receptor alpha-chain expression. Nat Immunol, 2008. 9(11): 1288-96.
5. Martin, C., H.D. Held, S. Uhlig, Differential effects of the mixed ET(A)/ET(B)-receptor antagonist bosentan on endothelin-induced bronchoconstriction, vasoconstriction and prostacyclin release. Naunyn Schmiedebergs Arch Pharmacol, 2000. 362(2): 128-36.
